# Thermoluminescence Response of Ge-Doped Cylindrical-, Flat- and Photonic Crystal Silica-Fibres to Electron and Photon Radiation

**DOI:** 10.1371/journal.pone.0153913

**Published:** 2016-05-05

**Authors:** A. Entezam, M. U. Khandaker, Y. M. Amin, N. M. Ung, D. A. Bradley, J. Maah, M. J. Safari, F. Moradi

**Affiliations:** 1 Department of Physics, Faculty of Science, University of Malaya, 50603 Kuala Lumpur, Malaysia; 2 Clinical Oncology Unit, Faculty of Medicine, University of Malaya, 50603 Kuala Lumpur, Malaysia; 3 Department of Physics, University of Surrey, Guildford, GU2 7XH, United Kingdom; 4 Department of Chemistry, Faculty of Science, University of Malaya, 50603 Kuala Lumpur, Malaysia; 5 Department of Biomedical Imaging, Faculty of Medicine, University of Malaya, 50603 Kuala Lumpur, Malaysia; North Shore Long Island Jewish Health System, UNITED STATES

## Abstract

**S**tudy has been made of the thermoluminescence (TL) response of silica-based Ge-doped cylindrical, flat and photonic crystal fibres (referred to herein as PCF-collapsed) to electron (6, 12 and 20 MeV) and photon (6, 10 MV) irradiation and 1.25 MeV γ-rays, for doses from 0.1 Gy to 100 Gy. The electron and photon irradiations were delivered through use of a Varian Model 2100C linear accelerator located at the University of Malaya Medical Centre and γ-rays delivered from a ^60^Co irradiator located at the Secondary Standard Dosimetry Laboratory (SSDL), Malaysian Nuclear Agency. Tailor-made to be of various dimensions and dopant concentrations (6–10% Ge), the fibres were observed to provide TL yield linear with radiation dose, reproducibility being within 1–5%, with insensitivity to energy and angular variation. The sensitivity dependency of both detectors with respect to field size follows the dependency of the output factors. For flat fibres exposed to 6 MV X-rays, the 6% Ge-doped fibre provided the greatest TL yield while PCF-collapsed showed a response 2.4 times greater than that of the 6% Ge-doped flat fibres. The response of cylindrical fibres increased with core size. The fibres offer uniform response, high spatial resolution and sensitivity, providing the basis of promising TL systems for radiotherapy applications.

## Introduction

Differentially angled X-ray external beam radiotherapy represents a key modality in the treatment of deep-seated malignant disease [1], with typically two or more beams used to provide the best possible outcome. In the delivery of dose to such tumours, the oncology teams are generally aided in this through use of computerized treatment planning (CTP) facilities, based on measured doses obtained in 3D water tanks using ionization chambers and sometimes diodes. Homogeneous target volume irradiations are sought, optimized to obtain minimized doses to surrounding healthy tissues. Such irradiations are applied in treatment of various cancers, including breast, brain, prostate, lung, gynecologic tumours, lymph and bladder [2], recent years seeing greater application of the precise fine beams of the stereotactic technique. For more superficial tumours single-beam accelerated electrons are a popular choice, albeit often without use of CTP. In line with all such developments, greater demands have been placed on the performance of the detectors used in calibration and beam monitoring, the establishment of one such detector being the focus of present interests.

Offering a number of advantageous features, including a detector size that has been well suited to conventional external beam radiotherapy, tissue equivalence, sensitivity and ease of use, commercially available phosphor-based thermoluminescence dosimeters (TLD) have found widespread applications in clinical dosimetry and quality assurance in medicine. LiF represents a particularly popular choice (with the commercial product identifier TLD-100), although the relatively large typical size (typically ~ mm) challenges its use in stereotactic radiotherapy and, being potentially hygroscopic, it fails to meet demands for detailed measurement of dose distributions within tissues [3]. As such, in recent years, a range of materials have been investigated, both to allow for entrance and exit dose evaluations, as well as radiation dosimeters intended for *in-vivo* evaluations [3–6]. Two of the more promising materials are SiO_2_-based optical fibres [7] and PCF-collapsed [8] (see below for details of the fabrication of these), offering lateral spatial resolution down to a few tens of microns [9], water and corrosion resistance capability and relatively low cost [10]. In addition, the fibres offer reproducibility and re-use without detriment to dose response [6]. The small size offers potential for the assessment of radiation dose in application of the fine beams of stereotactic radiotherapy as well as in measurements of dose within the body. In addition, its high sensitivity enables risk assessment even for low dose procedures.

In the absence of doping with well-chosen elements, the intrinsic defects of SiO_2_ fibres generally produce limited thermoluminescence. For the fibres used in optical communications, for effective light transport dopants are introduced into the silica in order to control the refractive index of the glassy host, producing the total internal reflection that is necessary. In regard to radiation dosimetry, these same dopants act as defect centres, trapping and storing electrons that have been excited through receipt of exposure to radiation. Accordingly, commercially available fibres doped with Ge have shown significant TL yield. Indeed, to-date investigations using Ge-doped silica have produced materials whose sensitivity to radiation are found to be superior to that of fibres doped with alternative elements such as ytterbium, erbium, aluminum, samarium, and neodymium [5–7]. It is fortuitous that due to the presence of dopants, irradiated commercially available optical fibres have been found to give rise to appreciable TL, albeit with limited success to-date in measuring doses down to the clinical diagnostic levels, the latter being of the order of a thousand fold less than the levels used in radiotherapy. Thus said, the potential of photonic crystal fibres (PCF) [8] have been demonstrated to be of use as a TL dosimeter at diagnostic radiology doses.

Recent detailed study of various parameters providing controlling roles in TL yield has pointed to fabrication of well-characterized Ge-doped silica-based fibres with practical applications beyond that of conventional radiotherapy [10]. However, in the present paper we focus our attention on investigations of the TL properties of tailor made PCF-collapsed and silica-based Ge-doped fibres for typical therapeutic applications. Studies have been carried out to investigate the TL response of these candidates dosimeters for megavoltage X-ray photons (6, 10 MV), MeV electrons (6, 12, and 20 MeV) and 1.25 MeV γ-rays, for a dose range of 0.1 to 100 Gy. The main purpose has been to obtain full performance characterization of these new candidate TL materials for radiotherapy dosimetry applications, due consideration being paid to PCF-collapsed and different dopant concentrations and dimensions of fibres.

## Material and Methods

### Fibre fabrication and Sample preparation

The fibres under investigation have been tailor-made at the Integrated Lightwave Research Group (ILRG) fibre pulling facility of the University of Malaya. In this study, two Ge-doped optical fiber preforms have been fabricated, referred herein as collapsed and uncollapsed preform. For each preform, a Suprasil F300 ultra-pure glass tube has been used as the substrate (Suprasil-300; Heraeus Holding GmbH, Hanau, Germany) and different dopant concentration (See Table 1) introduced in the fibre preform using a Modified Chemical Vapour Deposition (MCVD) process.

**Table 1:** Details of all types of silica based optical fibres used in study of various TL properties.

Purpose	Number of fibre category	Fibre types	Dimension (μm) ± 2 μm	Mass (mg) ± 0.02 mg	%Ge-dopant concentration (See in S2 Fig) ± 1%
Detailed TL studies	1	PCF-collapsed (fibre/core diameter)	270/38	1.50	8%
	2	Cylindrical (fibre/core diameter)	(270/42)	2.30	10
	3	Cylindrical (fibre/core diameter)	(241/33)	1.30	10
	4	Flat	750×154	6.40	10
	5	Flat	750×154	6.40	6
Gross TL sensitivity studies	Total of 18 fibre dosimeters	Cylindrical (fibre/core diameter)	100/14	1.00	6% & 10% Ge
			120/18	1.00	
			241/33	1.30	
			270/42	2.30	
			362/60	2.80	
			483/75	3.50	
			604/82	4.10	
			620/85	4.80	
			750/102	5.50	
	Total of 12 fibre dosimeters	Flat	180× 36	1.50	6% and 10% Ge
			270× 52	2.60	
			350× 72	3.10	
			510×100	4.00	
			620×124	5.20	
			750×154	5.80	

The Ge-doped collapsed preform is used to fabricate the flat and cylindrical fibers. The cylindrical fibers were drawn from a large glass tube, obtaining the desired optical fiber size at a temperature of around 2000°C, controlling the preform feed-rate and drawing-speed, also considering the simplified mass conservation law:

$$A_{1}v_{f}=A_{2}v_{d}$$ (1)

where *A*_1_ and *A*_2_ are the cross-sectional areas of preform and fiber while *v*_*f*_ and *v*_*d*_ are the feeding and drawing-speed, respectively.

The flat fibres have been fabricated in a similar way to the cylindrical fibre. However, a critical difference is the use of vacuum pressure (to-date, of 10 kPa) applied from the top of the hollow glass tube in order to collapse the tube into a flat shape. As a result, the internal walls coming into contact with each other, subsequently fusing prior to cooling, the shape of the pulled cylindrical fibre being converted into a flat one.

**Fig 1:**
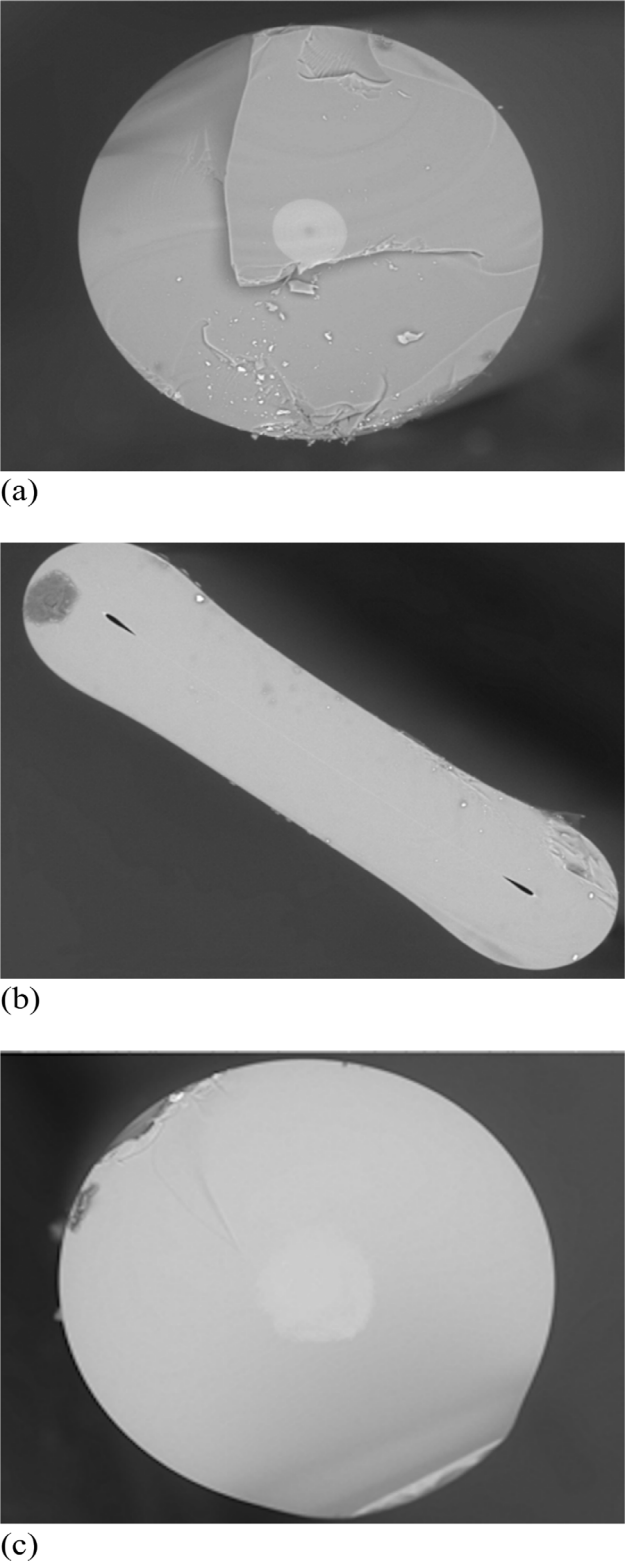
Photomicrograph view. Fig show the cross-sectional Photomicrograph of: (a) 270 μm Ge-doped cylindrical fibre with a core size of 42 μm, (b) 750 μm Ge-doped flat fibre, and (c) PCF-collapsed (see Table 1 for full details).

On the other hand, the Ge-doped preform tube that has been left in its hollow array of holes form, referred to as uncollapsed-preform [11]. This Ge-doped uncollapsed-preform is used to fabricate the PCF by using the stack-and-draw method [8]. The uncollapsed preform is first drawn into 1.26 mm diameter capillary canes. The capillaries are then cut into 30–40 cm length, fused from one end, cleaned, and stacked into a 25 mm /19 mm outer/inner diameter Suprasil F300 tube. The central capillary is later replaced with an undoped-silica rod to form the solid-core PCF. The PCF preform is then drawn into about 1–2 mm diameter PCF cane and subsequently the cane is re-pulled into a standard fiber size of 125 ± 5 μm. Later, from the same PCF cane, a different version of PCF is drawn down by applying a vacuum pressure from the top of the PCF cane during the fabrication process, causing the entire array of holes in the PCF to be fully collapsed as shown in Fig 1(C). As a results, it generates additional defects and thereby, greater TL response.

In regard to the effective atomic number, *z*_*eff*_, this was determined using the Mayneord equation:

$$z_{eff}={\left(\;a_{1}z_{1}^{m}+\;a_{2}z_{2}^{m}+a_{3}z_{3}^{m}+\cdots \;\ldots .+a_{n}z_{n}^{m}\;\right)}^{\frac{1}{m}}$$

where *a*_1_, *a*_2_, … ….,*a*_*n*_ are the weight fraction contributions of each element in the optical fibres to the total number of electrons in the mixture. The value of m adopted for photon practical purposes is 2.94 [12], the calculated effective atomic number then being in the range 14.89 to 16.09. Account of the effective atomic number is needed in accurate determination of radiation dose to the irradiated tissues; as an instance, *Z*_*eff*_ for soft tissues is 7.42 while *Z*_*eff*_ for bones is in the range 11.6–13.8 [13].

A total of 30 different optical fibres (different in shape and dopant concentrations) were fabricated for study of TL sensitivity (Table 1). Pilot investigation of the TL response was made for all thirty dosimeters, the outcome leading to limitation of the full study to the most sensitive of these (those offering the greatest TL yield per unit dose), namely PCF-collapsed, two different core sizes of Ge-doped cylindrical optical fibre and two flat fibres (Fig 1). These selected fibres were found to offer the most promising TL yield.

The fibre samples were first cleaned using methyl alcohol and then cut into 5 ± 1 mm segments using an optical fibre cleaver (Cl-03 Max, ILSIN, Korea) and diamond-cutter (S90R, Thorlabs). The choice of sample length concerned ease of handling in TL readout. The weight of each fibre was determined using an electronic balance (Mettler Toledo, Switzerland), used for TL normalization purposes to account for the mass variations of the fibre segments types. The samples were handled using vacuum tweezers to avoid surface abrasion and contamination, both of which could affect TL response. In addition, the fibers were encased in small plastic discs or envelopes (all radiolucent) for ease of handling. Table 1 shows detailed information on the physical properties of the prepared samples.

#### Annealing procedure

Prior to irradiation, the samples were annealed to remove the background signal. The annealing procedure was performed with the samples in a ceramic holder, annealed in an oven (Lindberg/Blue M, USA) at a temperature of 400°C for one hour [14]. After heating, the samples were slowly cooled to room temperature over a period of 24 hours. All fibres were then kept in small black plastic bags for routine storage and easy handling.

#### Irradiation of fibres

The prepared samples were irradiated by a range of radiations: accelerated electron energies (6, 12, and 20 MeV), megavoltage x-ray photons (6, 10 MV) and high energy γ-rays (mean energy 1.25 MeV). The electrons and x-rays were delivered by a Varian Model 2100C linear accelerator (LINAC) located at the University of Malaya Medical Centre. A dose range of 0.1 Gy to 100 Gy was delivered to the samples using a source to surface of sample distance (SSD) of 100 cm. A solid-water^TM^ phantom (Gammex, USA) (30 cm length × 30 cm width) was used to provide for the typical full-scatter calibration condition. The γ-rays were delivered from a ^60^Co irradiator, the samples being irradiated to a dose of 100 cGy, the irradiator being located at the Secondary Standard Dosimetry Laboratory (SSDL), Malaysian Nuclear Agency. A perspex (Polymethyl methacrylate, PMMA) phantom with dimension of (30 × 15 × 30) cm^3^ was used in delivering full scatter conditions to the samples at the points of γ-ray irradiation.

#### TL readout system

The TL yield of the irradiated fibres were readout using a Harshaw 3500 TLD reader, readings being carried out under nitrogen (N_2_) gas atmosphere. The readout parameters were set up to ensure optimal data acquisition and radical sweep-out of any residual signal: preheat temperature of 50°C for 10 s, an acquired temperature rate of 40°C/s, an acquisition time of 13.33 s and maximum anneal temperature of 400°C.

### Characterization of irradiated fibres for TL properties

#### Reproducibility and linearity

The electron LINAC was set to deliver a 6 MV X-ray beam with a field size of 10 cm. The fibre dosimeters were placed within the solid-water^TM^ phantom on the central axis, at the isocentre at 1.5 cm depth, a depth at which the maximum dose is delivered to the detectors. A minimum of 10 segments of PCF-collapsed and each type of Ge-doped optical fibres were irradiated by 6 MV X-ray beams for each dose, allowing assessment of statistical variation and reproducibility. Linearity and sensitivity measurements were performed for a range of typical doses delivered to the patient, with conventional fractionated doses of 25, 60, 120, 300 and 550 cGy for the different types of fibre. The reproducibility of the fibre types was determined using the resultant data. It should be noted that for each type of fibre we fabricated fibres of some 10 to 20 meter length. A number of individual dosimeters (segments) were prepared, taken randomly from different parts of the fibre length, in order to investigate the quasi uniform distribution of Ge-dopant in the fibres. Further investigation of reproducibility was also carried out for 6-, 12- and 20-MeV electron energy at a dose of 100 and 120 cGy for two selected fibers (of 42 μm core size cylindrical) and PCF-collapsed. Detailed information on the reproducibility for the selected cylindrical fibres (42 μm core size cylindrical and PCF-collapsed) is presented in Table 2. Additionally, Table 3 shows similar standard deviation (SD) values compared to the standard TLD-100 and TLD-700 dosimeter [15–17], and this fact confirms the accuracy and robustness of the studied fiber dosimeters.

**Table 2:** Dose response reproducibility using electron and photon beams.

Source	Dose (cGy)	Number of studied segments from one type of fibre	Charge (nC)					
			Mean		SD		SD in %	
			42 μm fiber	PCF-collapsed	42μm fiber	PCF-collapsed	42μm fiber	PCF-collapsed
6 MV photons	25	6	6.90	15.80	0.39	0.48	5.65	3.06
	65	6	8.90	21.36	0.43	0.40	4.83	1.89
	120	5	12.08	29.00	0.72	1.72	5.96	2.98
	300	5	23.82	60.30	0.90	1.27	3.77	2.13
	550	8	46.57	109.30	1.95	0.90	4.18	0.83
6 MeV electrons	100	7	12.10	29.80	0.69	0.53	5.70	1.80
12 MeV electrons	100	7	12.55	30.02	0.15	0.33	1.19	1.10
20 MeV electrons	100	8	12.60	29.65	0.32	0.62	2.53	2.10
	120	6	15.02	35.62	0.72	1.15	5.80	3.23

**Table 3:** Comparison of SD values of clinically used dosimeters with the studied silica based dosimeter.

Source	Detector	SD% (mean)	References
4MV X-ray beam	TLD 100	3.5%	Luxton, (1999) [15]
4MV X-ray beam	TLD 100	4%	Veneziani, (2010) [16]
X-ray beams in the range of effective energies between 33 and 116 keV	TLD 100	4%	Miljanic, (2002) [17]
X-ray beams in the range of effective energies between 33 and 116 keV	TLD 700	6%	Miljanic, (2002) [17]
6 MV Photon, 6–20 MeV electron	Optical Fiber	4.8%	This work
6 MV Photon, 6–20 MeV electron	PCF-collapsed	2.12%	This work

#### Energy dependency

**Fig 2:**
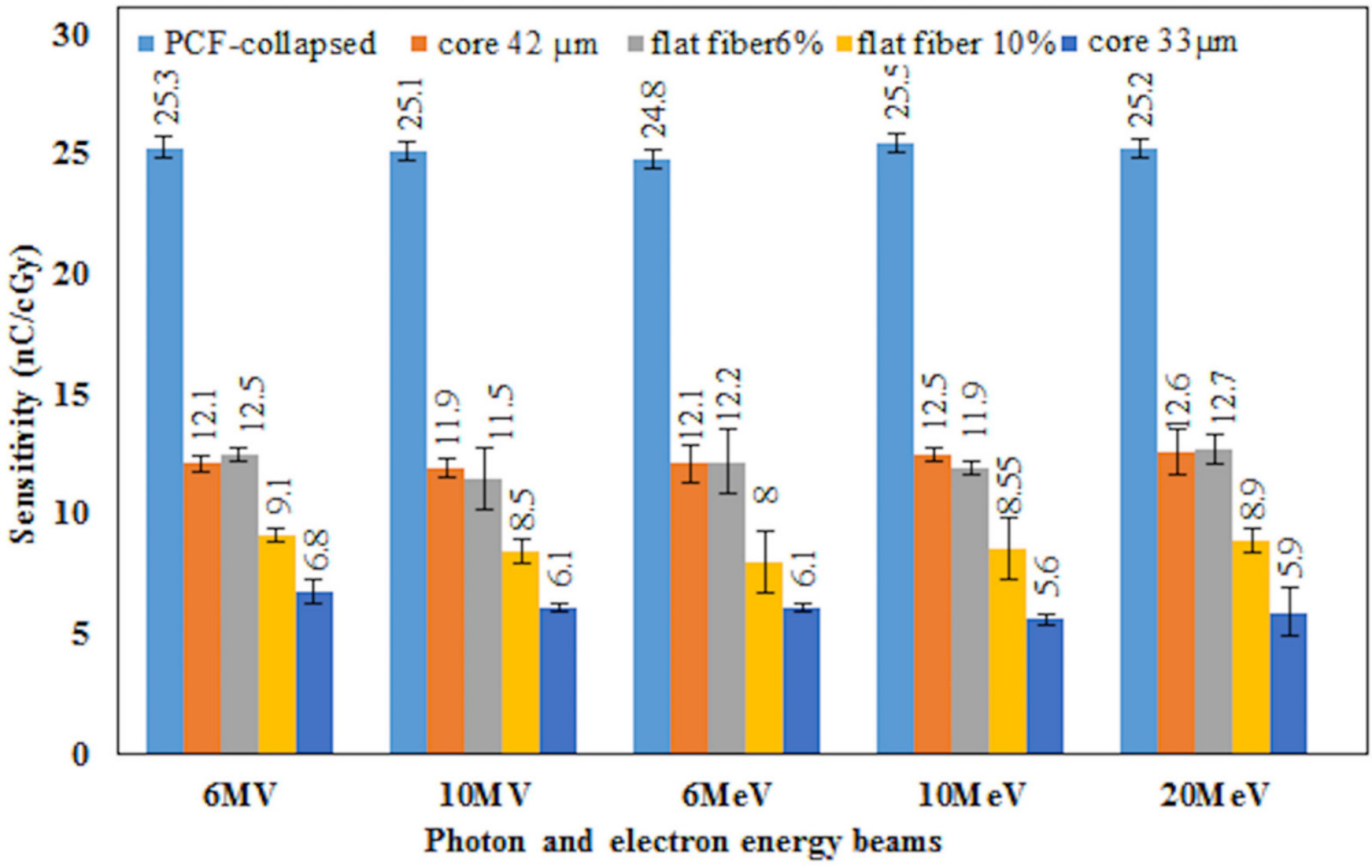
Sensitivity of fibres of PCF-collapsed, comparison of sensitivity of 42 μm &33 μm core sizes cylindrical and flat fibres (10% & 6% Ge-dopant) for different energies is shown.

The sensitivity of the detectors is defined as the ratio of readout value to the given dose. The sensitivity of the fibres were obtained for two photon energies, 6- and 10-MV, and three electron energies, 6-, 12- and 20-MeV. For two selected fibres (42 μm core size cylindrical) and PCF-collapsed further irradiation was carried out for the 1.25 MeV γ-ray energy. Measurement for five fibre segments for each fibre type was carried out at 100 cm SSD and 10 × 10 cm^2^ field size. Fig 2 represents the results of sensitivity of the studied PCF-collapsed and optical fibres by labeling with respective numerical values (for 42 μm core cylindrical and 6% flat fiber). In addition, Table 4 represents the mean sensitivity of the studied fibres, showing the PCF-collapsed sensitivity to be some 2.4 times greater than 6% Ge-doped flat fiber (and 4 times greater than 10% Ge-doped flat fibre), the overall sensitivity for the 42 μm core size fibre being slightly greater than 6% Ge-doped flat fibre.

**Table 4:** The mean sensitivity of the studied fibres.

Source	PCF-collapsed	Core 42 μm	Core 33 μm	Flat fiber 6%	Flat fiber 10%
6MV photon	25.30	12.08	12.50	9.10	6.85
10MV photon	25.11	11.96	11.50	8.50	6.13
6MeV electron	24.80	12.10	12.23	8.00	6.10
10MeV electron	25.49	12.55	11.90	8.55	5.67
20MeV electron	25.20	12.6	12.77	8.90	5.94
**Mean**	**25.18**	**12.26**	**12.10**	**8.61**	**6.13**

#### Field size dependency

**Fig 3:**
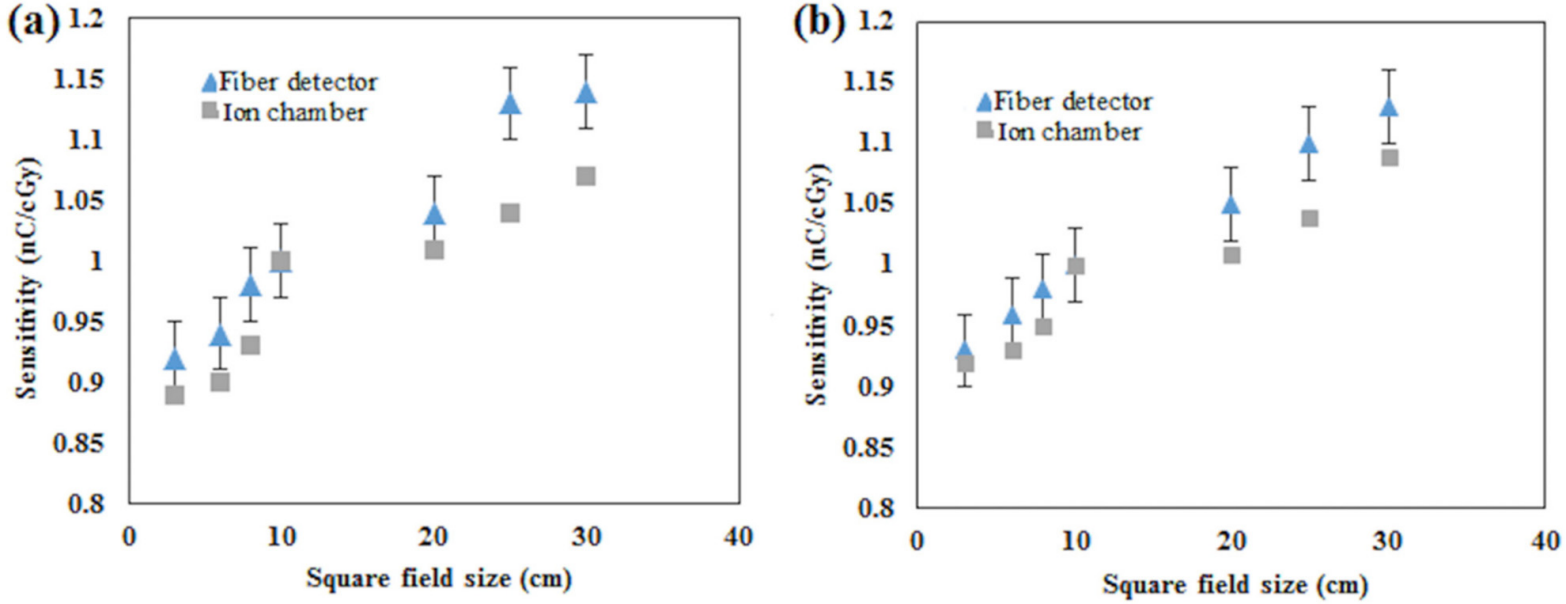
Sensitivity dependency of the 42 μm core-size fibre on field size. Sensitivity dependency on field size measured for (a) 6MV and (b) for 10MV X-rays of the 42 μm core-size fibre.

**Fig 4:**
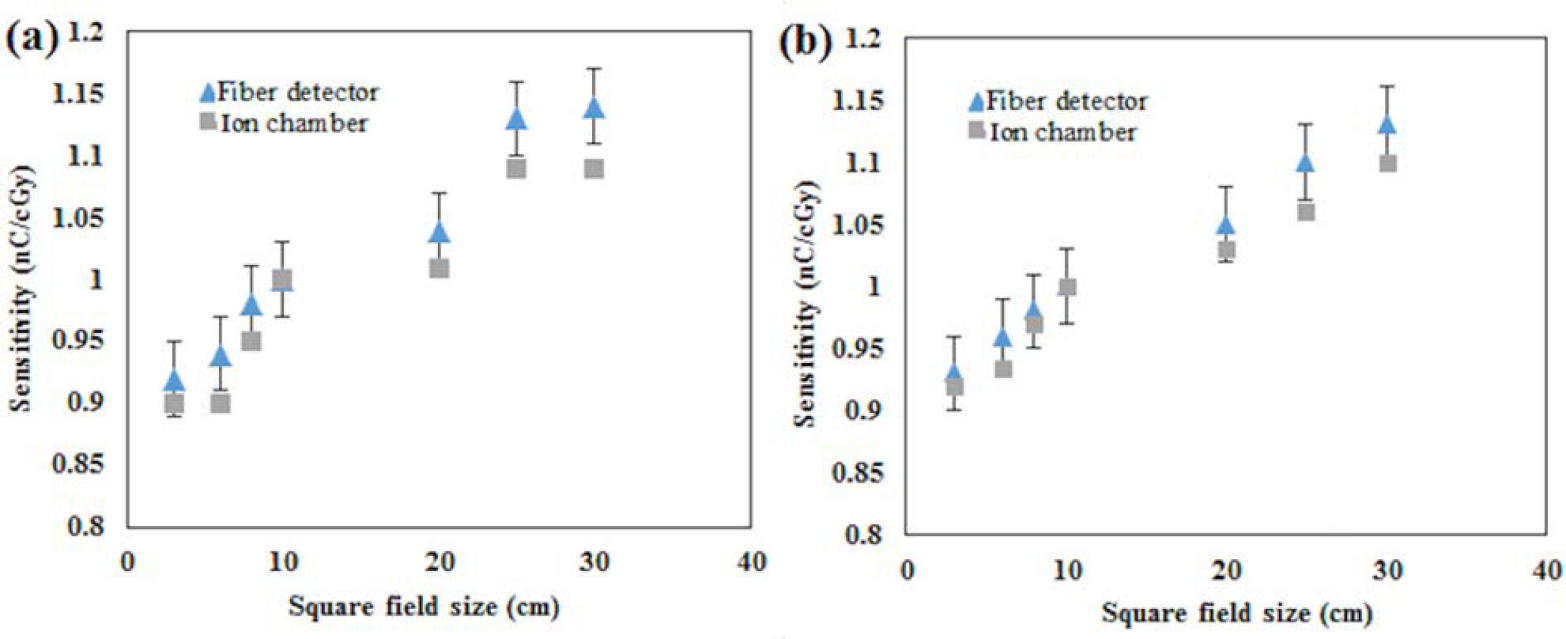
**Sensitivity dependency of the PCF-collapsed on field size**, the Sensitivity dependency of the PCF-collapsed on field size was measured for (a) 6MV and (b) for 10MV X-rays.

Dependency on the field size for the most sensitive samples PCF-collapsed and the 42 μm core size fibre was determined for photons produced at 6- and 10 MV and field sizes of 3, 6, 8, 10, 20, 25 and 30 cm. The detectors were placed on the surface of the phantom, and then covered with a 1.5 cm solid-water^TM^ layer to locate the fibres at the depth of maximum dose build-up. The accelerator was set up to deliver the maximum dose of 100 cGy for square field of 10 cm size. The field size dependency was compared with the output factor for the maximum dose. The output factor was measured with an ionization chamber. For each field size, measurement was carried out for five fibre segments of the 42 μm core size fibre, the results being normalized for all field sizes using the obtained value for 10×10 cm^2^ field size (see Figs 3 and 4).

#### Angular dependency

**Fig 5:**
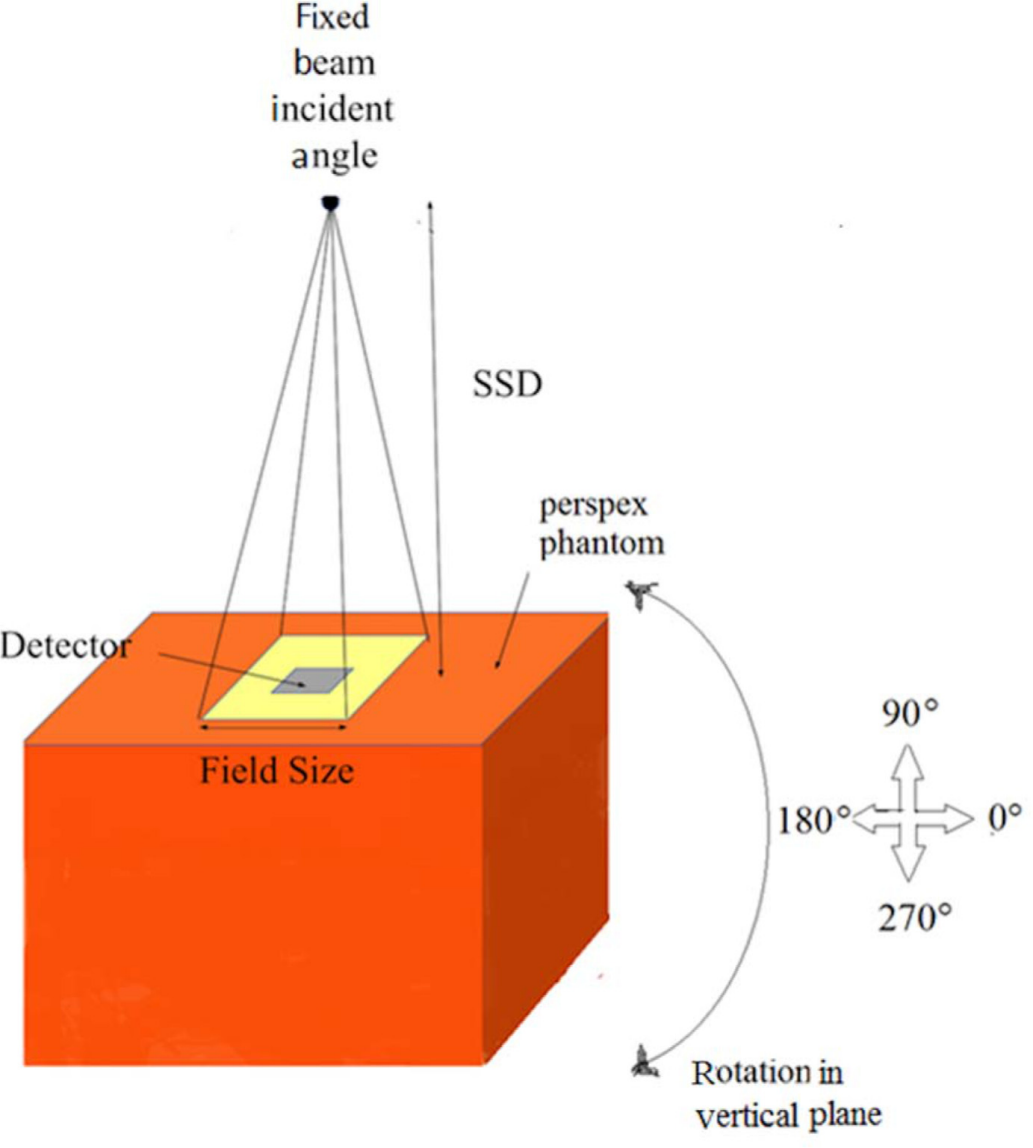
Schematic illustrations of the setup for the angular dependency study. The position of the detector and the irradiator is shown in the fig.

Angular dependency evaluation was carried out by exposing the PCF-collapsed and 42 μm core-size fibre to γ-radiation at a dose of 100 cGy using the ^60^Co irradiator. For each chosen angle of irradiation, and as before, a total of five fibres were placed within a perspex (methyl methacrylate) phantom such that the maximum dose was delivered to the detectors, use being made of 100 cm SSD and field size 10 × 10 cm^2^, the phantom being rotated from 0 to 360° in incremental steps of 30°. Fig 5 represents the schematic illustrations of the measurement setup.

## Results and Discussion

### Linearity and reproducibility

**Fig 6:**
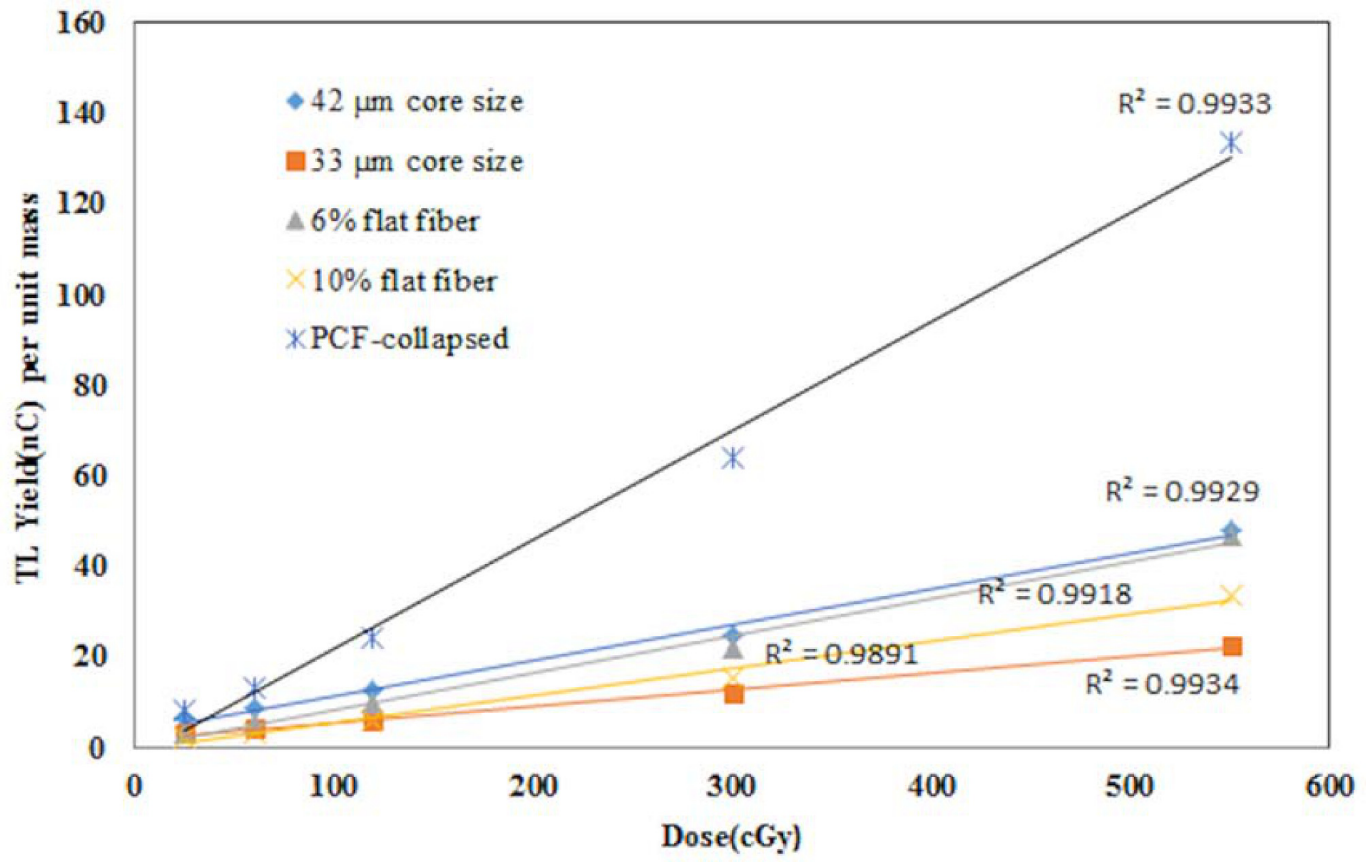
Comparison of dosimetric response for PCF-collapsed cylindrical and flat fibres. Figure shows the dosimetric response for PCF-collapsed.10% Ge-doped (33 & 42 μm core diameter cylindrical fibres) and flat-fibres (6 & 10% Ge-doped), irradiated with 6 MV photons for doses from 25–550 cGy.

Fig 6 compares linearity of response for the cylindrical, flat and PCF-collapsed fibres under study, exposed to 6 MV X-rays for doses from 0.25 Gy to 5.50 Gy, 6 MV photons being popularly used in external beam radiotherapy. Regardless of the type, shape and dopant concentration, a regression coefficient R^2^ > 0.9898 was obtained (See in S1 Fig), supporting the findings of Espinosa [6] for a commercial silicon-dioxide optical-fibre, Abdulla [4] for a Ge-doped optical-fibre and Amouzad [8] for PCF-collapsed. The results provide a step forward, compared to the use of TLD-700 in the IAEA/WHO TLD postal programme, non-linearity being the dominant source of uncertainty therein [18]. Flat fibres, 6% dopant concentration, produced a superior TL response to that of 10% flat fibres. For change in TL yield per unit mass, cylindrical 10% dopant fibres of 42 μm core-size proved superior to 10% dopant 33 μm core-size fibre of identical dopant concentration. Least-squares fitting showed the response of PCF-collapsed to be about 4 times greater than the 10% dopant flat fibre. The 42 μm core fibre (270 μm cladding diameter) provided the second greatest response (R^2^ = 0.993) after the PCF-collapsed. Thus, PCF-collapsed and 42 μm core-size cylindrical fibers were selected for further study of reproducibility, for 6-, 12-, and 20-MeV electron energies, with a dose of 100 cGy; details are as in Table 2.

From Table 2 it is apparent that despite the absence of prior individual calibration the reproducibility of the fibre dosimeters’ response was always better than 6%, while the PCF-collapsed shows reproducibility to be always better than 4%, confirming the uniform distribution of Ge-dopant in the fibres. The stability of TLD reader output was another factor affecting reproducibility. For the same samples, during the course of a day the TLD reader output accounted for of the order of 3% variation.

**Fig 7:**
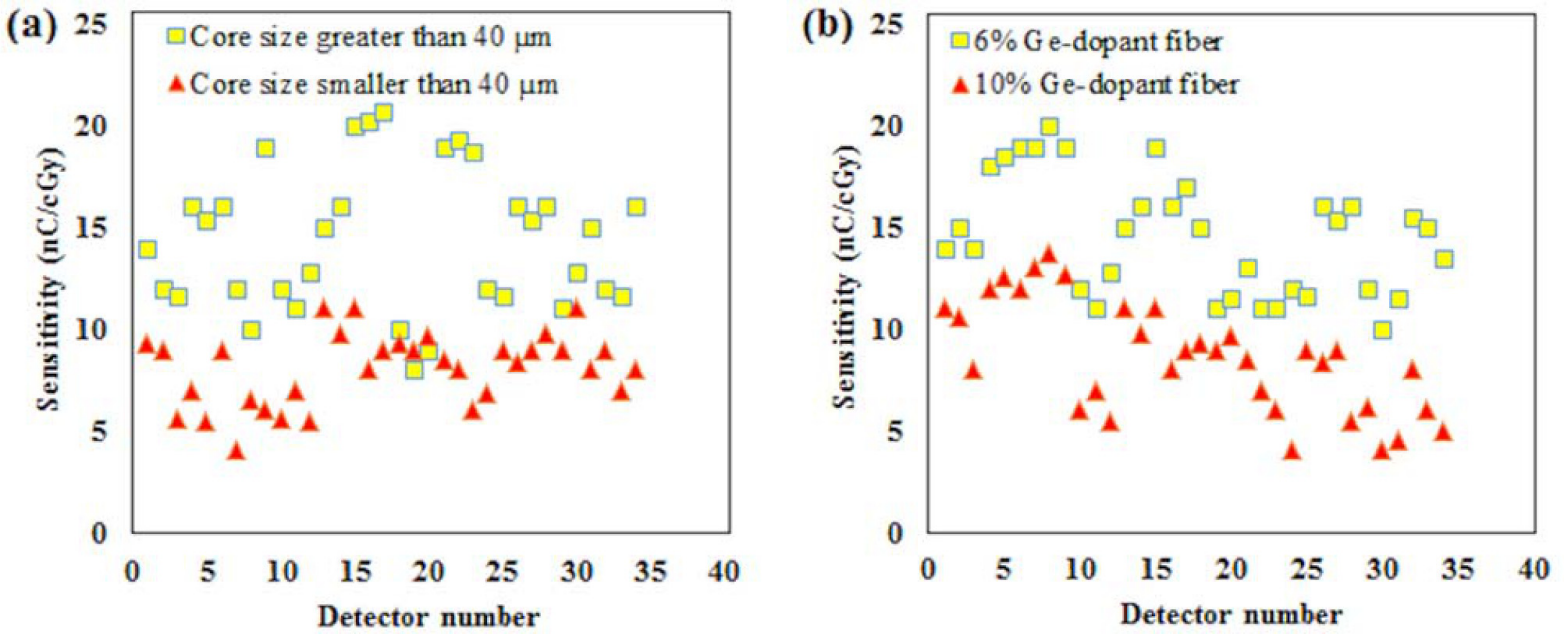
TL sensitivity for different core size and Ge concentration detectors. TL sensitivity of detectors with respect to: (a) Core diameter and; (b) Ge concentration.

Beside the PCF-collapsed, a total of some 38 optical fibres of each size and dopant concentrations were fabricated and studied herein, a 6 MV photon beam delivering a dose of 100 cGy. Their sensitivity with respect to dopant concentration and core diameter are shown in Fig 7(A) and 7(B) respectively. Measurement was performed for 1 to 3 fibre segments from each type of fibre. Fig 7(A) and 7(B) indicate the smaller dopant concentration (6% Ge) and larger core size (> 40 μm) to provide an overall greater TL sensitivity. This result supports those of [19, 20].

**Fig 8:**
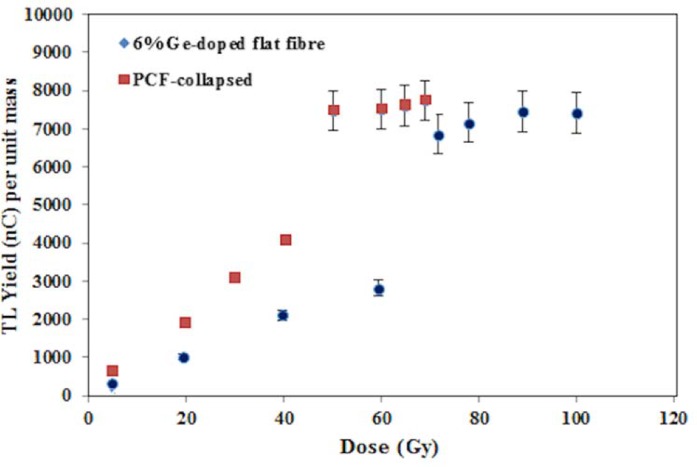
TL response of the 6% Ge-doped flat fibre and PCF-collapsed for high dose range. The fig compares the response of of the 6% Ge-doped flat fibre and PCF-collapsed for high dose range.

Again with 6MV photon irradiation, the TL response of the6% Ge-doped flat fibers and PCF-collapsed were investigated for an elevated dose range (5.50–100 Gy). Fig 8 shows the TL yield to gradually increase for doses up to 64 Gy and 43 Gy (R^2^ > 0.99), with subsequent sharp increase for doses in the ranges 64 to 70 Gy and 43 to 74 for Ge-doped flat fibre and PCF-collapsed respectively before saturation, presumably with all trapping centres then occupied.

### Energy dependency

**Fig 9:**
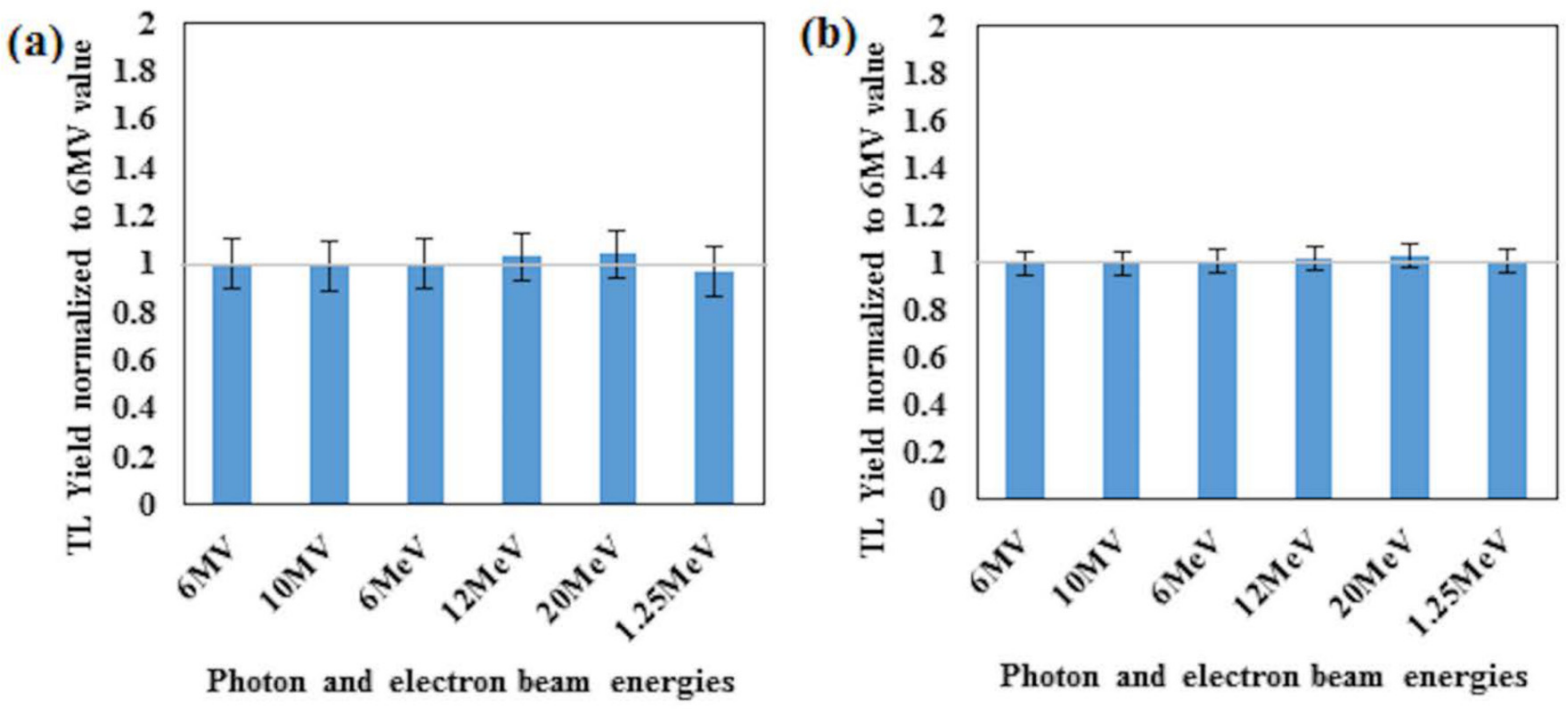
The dependency of energy for fibres. Energy dependency of (a) 42 μm core sizes, and (b) PCF-collapsed is shown in the figure.

Fig 2 shows the sensitivity of fibre dosimeters exposed to X, and electron radiation. The dependency on energy of the beam is minimal, in all cases other than the 33μm core size fibre being within the size of the 1 σ error bars; for the 33 μm core size fibre the response is in agreement to within 2 σ. Numerical details (Table 5 and Table 6) and the plot of the dependency on the energy (Fig 9(A) and 9(B)) for the 42 μm core size optical fibre and PCF-collapsed are presented, respectively.

**Table 5:** Dependency on energy for fibre of 42 μm core size.

Parameter	Co-60 (γ-rays)	Photon (X-rays)		Accelerated electrons		
**Energy**	1.25 MeV	6 MV	10 MV	6 MeV	12 MeV	20 MeV
**Sensitivity [nC/cGy]**	11.66	12.08	11.96	12.10	12.55	12.60
**SD [%]**	4.5	1.5	2.8	4.7	1.1	3.2

**Table 6:** Dependency on energy for PCF-collapsed.

Parameters	Co-60 (γ-rays)	Photon (X-rays)		Accelerated electrons		
**Energy**	1.25 MeV	6 MV	10 MV	6 MeV	12 MeV	20 MeV
**Sensitivity [nC/cGy]**	29.10	29.15	29.35	29.80	30.02	29.65
**SD [%]**	2.8	1.5	2.4	1.8	1.1	2.1

The 42 μm core-size fibre detectors exposed to 10 MV X-rays have sensitivity closely similar to that using irradiations at 6 MV while the sensitivity for the PCF-collapsed exposed to 6 MeV electrons is close to that obtained at 12 MeV. Conversely, greater sensitivity is obtained for 20 MeV electrons compared to that at 6 MeV, possibly due to detection of neutrons that have been generated and perhaps due to the greater presence of low energy backscattered electrons [21]. The sensitivity of PCF-collapsed and the 42 μm core-size fibre exposed to 1.25 MeV γ-rays are less than that obtained at all other photon and electron energies.

### Field size dependency

For 6 and 10 MV X-rays, Fig 3A and 3B show the relative sensitivity of the 42 μm core-size optical fibre with output factor at the maximum dose as obtained from an ion chamber. Fig 4A and 4B show the same relative sensitivity for the PCF-collapsed, with both detectors showing a trend towards increase as the field size increases. This is expected due to the increase in backscattered radiation from the phantom and radiation contamination. The radiation contamination was mainly contributed to by the scattered radiation from the LINAC flattening filter, small compared to the scattered radiation arising from field size [22]. Good agreement has been observed between both fibre detectors and ion chamber (average difference of 1%). This indicates that the sensitivity dependency of both detectors with respect to field size follows the dependency of the output factors. A similar result was reported by [23] using a MOSFET detector. Furthermore, no significant difference for field size dependency was observed when the 42 μm core-size optical fibre and the PCF-collapsed were exposed to 10 MV X-rays.

### Angular dependence

**Fig 10:**
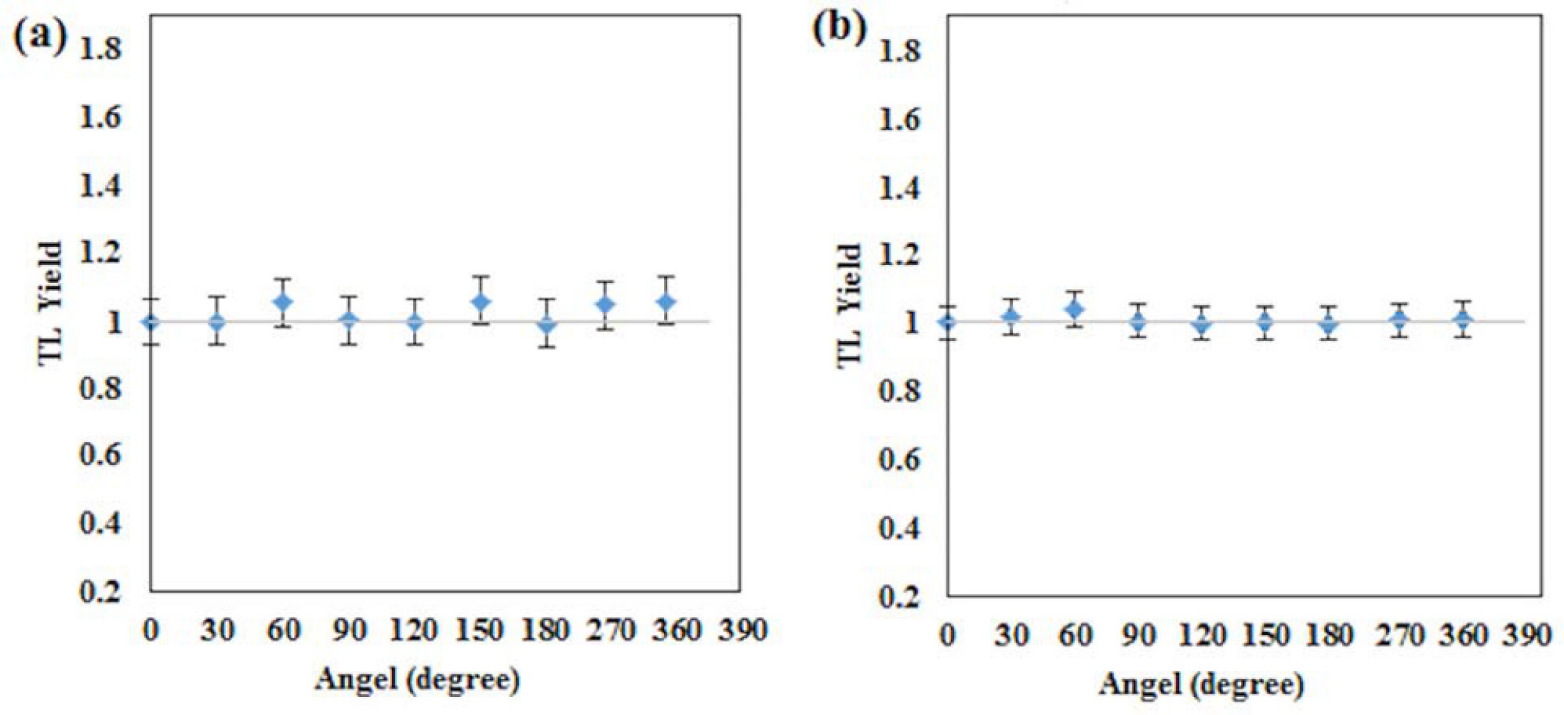
The normalized TL Yield of angular dependency at 0 degree. The fig indicate that the fibre optic detectors have minimal (<1%) angular dependency For (a) 42 μm core sizes, and (b) PCF-collapsed fibres.

Both PCF-collapsed and Ge-doped fibre of 42μm core size were found to have no measurable angular dependency for γ-radiation at gantry angles, from 0° to 360° (see the normalized TL Yield for angular dependency at 0 degree in Fig 10). Beside the possibility of asymmetric doping within the fibres, it is possible that microcrystalline structures could produce an orientational effect. Literature shows other small dosimeters such as diodes, diamond [24, 25] and MOSFET detectors [23] are dependent on angular position of irradiation.

## Conclusions

We have reported on the dosimetric response of tailor-made Ge-doped SiO_2_ fibres exposed to electrons, X- and γ-radiation, for doses from 0.1 Gy to 100 Gy. The fibres have been characterized for TL sensitivity, reproducibility and dependency on radiation field size, energy and angle. The fibres are linear with radiation dose up to 64 Gy, at R^2^ > 0.980 (at the 95% confidence level) but plateau for doses ≥70 Gy as a result of occupied defects saturation. All studied fibres offer reproducible response to within 1 to 5% for the various beams, the fibre with 42 μm core-size exposed to 12 MeV electrons showing ~1% reproducibility. In regard to field size dependency, the output factors measured with the fibres agree to within 1.5% with output factors measured via an ion chamber.

Among all the dosimeters studied herein, the PCF-collapsed response is some 2 times greater than the most sensitive cylindrical optical fibre detector (42 μm core size). The 6% Ge concentration fibre provided the greater response, the larger core-size fibre providing the greatest response after PCF-collapsed. With respect to the range of energies investigated the TL yield reveals no measurable variation to within 2σ. Similarly, no measurable variation was observed with change in angulation of the ^60^Co beam, offering advantage over several other small detectors, including diamond and MOSFETs.

## Supporting Information

S1 Fig Dose versus yield for 42 μm core size cylindrical fiber. The graph shows the linearity of dose versus yield for 6 MV beam.
(DOCX)

S2 Fig The SEM-EDX analysis. The figure shows the concentration of the Ge (5.9–9.8 mol%) in the studied fibres.
(DOCX)
