# Supplementary material for: Thermoluminescence Response of Ge-Doped Cylindrical-, Flat- and Photonic Crystal Silica-Fibres to Electron and Photon Radiation
Source: PLoS One. 2016 May 5;11(5):e0153913. doi: 10.1371/journal.pone.0153913 (PMC4857927; doi:10.1371/journal.pone.0153913)
Supplement: S1 Fig — The graph shows the linearity of dose versus yield for 6 MV beam. (DOCX) [file pone.0153913.s001.docx]

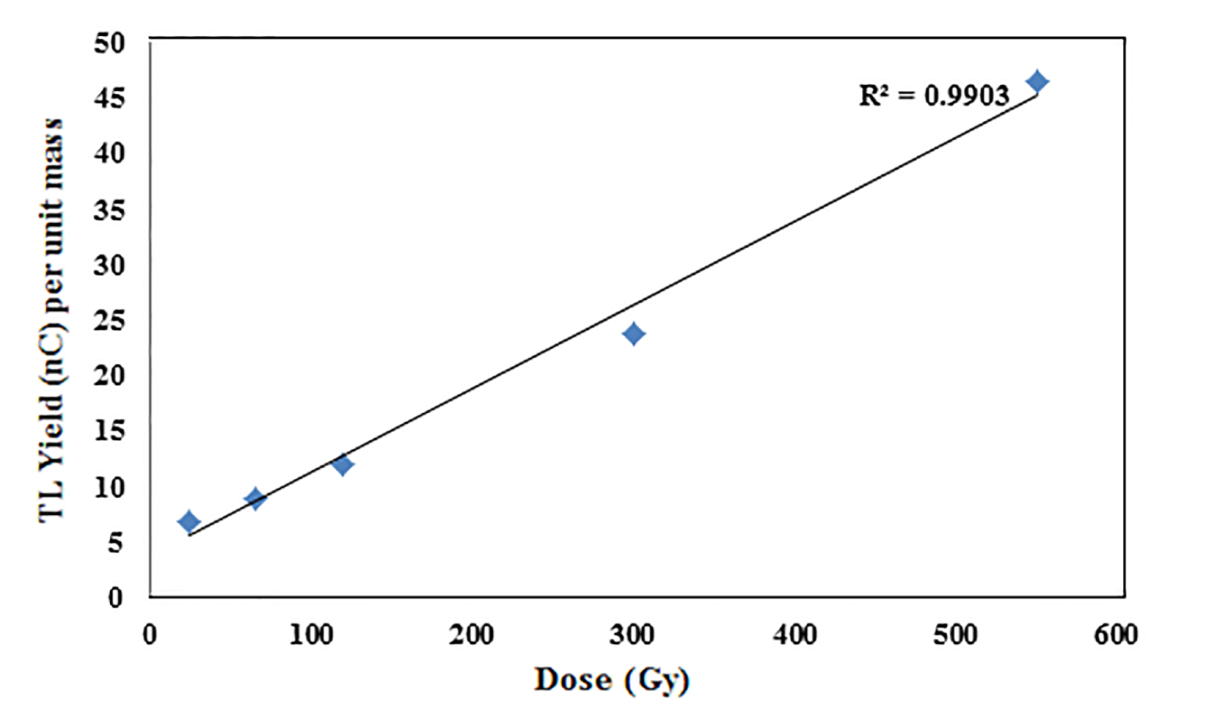


**S1 Fig. Dose versus yield for 42 µm core size cylinderical fiber.** The graph shows the linearity of dose versus yield for 6 MV beam.
