# Supplementary figures and images for: Thermoluminescence Response of Ge-Doped Cylindrical-, Flat- and Photonic Crystal Silica-Fibres to Electron and Photon Radiation
Source: PLoS One. 2016 May 5;11(5):e0153913. doi: 10.1371/journal.pone.0153913 (PMC4857927; doi:10.1371/journal.pone.0153913)

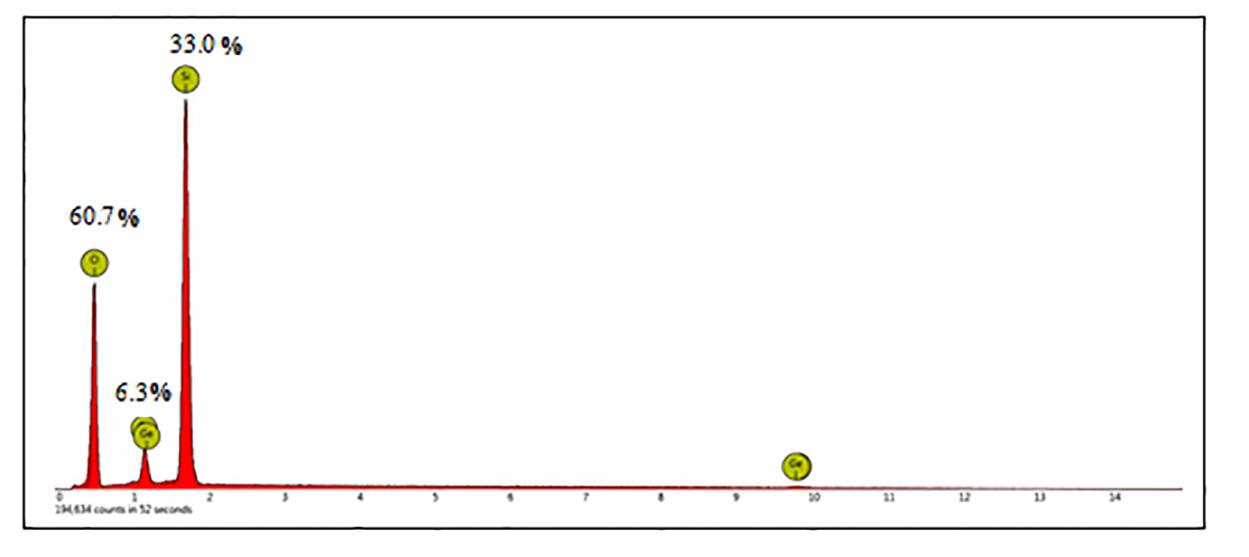


**S2 Fig. The SEM-EDX analysis.** The figure shows the concentration of the Ge (5.9-9.8 mol%) in the studied fibres.

Supplement: S2 Fig — The figure shows the concentration of the Ge (5.9–9.8 mol%) in the studied fibres. (DOCX) [file pone.0153913.s002.docx]
